# Insulinoma Presenting With Postprandial Hypoglycemia in a Pregnant Woman With MEN-1

**DOI:** 10.1210/jcemcr/luac015

**Published:** 2022-11-30

**Authors:** Anna J Wood, Vidya Kasireddy, Sridhar Chitturi, John P Walsh

**Affiliations:** Department of Endocrinology, Royal Darwin Hospital, Casuarina, Northern Territory 0810, Australia; Menzies School of Health Research , Charles Darwin University, Casuarina, Northern Territory, Australia; Department of Endocrinology, Royal Darwin Hospital, Casuarina, Northern Territory 0810, Australia; Department of Endocrinology, Royal Darwin Hospital, Casuarina, Northern Territory 0810, Australia; School of Medicine, Flinders University, Northern Territory Medical Program, Tiwi, Northern Territory, Australia; Department of Endocrinology and Diabetes, Sir Charles Gairdner Hospital, Nedlands, Australia; Medical School, University of Western Australia, Crawley, Australia

**Keywords:** multiple endocrine neoplasia type 1 (MEN1), insulinoma, hypoglycemia, pregnancy

## Abstract

Insulinomas are rare insulin-secreting tumors of pancreatic origin that cause hypoglycemia and can be associated with multiple endocrine neoplasia type 1 (MEN1). While rare, they are the most common cause of hypoglycemia related to endogenous hyperinsulinism. A 28-year-old woman with known MEN1 presented with postprandial hypoglycemia in the second trimester of pregnancy. Prior to her presentation she was known to have several pancreatic neuroendocrine tumors that had been stable on serial imaging, but no history of hypoglycemia. She was managed with dietary intervention during pregnancy and gave birth to a healthy baby at 37 weeks’ gestation. After pregnancy, hypoglycemia initially resolved, but then recurred at 8 months post partum. Magnetic resonance imaging showed several pancreatic neoplasms with the largest lesion measuring 29 mm in the pancreatic tail, unchanged from previous imaging. After localization with a selective arterial calcium stimulation test, the patient underwent successful distal pancreatectomy with resolution of symptoms. This case is unusual in that her initial presentation was during pregnancy, she had predominantly postprandial rather than fasting hypoglycemia, and her symptoms remitted for several months after delivery. Key learning points are to have a low index of suspicion for an insulinoma when there is a history of MEN1 and the need for a pragmatic approach to diagnosis and treatment during pregnancy.

Pregnancy is considered a physiological state of insulin resistance, hence an insulinoma first presenting as hypoglycemia in pregnancy is rare. While insulinomas presenting in pregnancy are described in the literature [1, 2], the majority of these cases present in the first trimester (before peak insulin resistance) or post partum. We report the case of a young woman who presented with symptomatic hypoglycemia in her second trimester of pregnancy, which was subsequently confirmed to be due to an insulinoma. Interestingly, her symptoms initially improved post partum. In addition, because she presented during pregnancy, reference ranges for performing a mixed-meal test were unclear and the safety of investigations and treatments were either not well established or with limited safety data. This case highlights some of the difficulties both in making an accurate diagnosis of the cause of hypoglycemia during pregnancy and safely treating women during pregnancy.

## Case Presentation

Mrs A is a 28-year-old woman who presented with hypoglycemia at 18 weeks’ gestation (gravida 2 para 1), on a background of recently diagnosed gestational diabetes and known multiple endocrine neoplasia type 1 (MEN1) with an S38F mutation in the menin gene. Consistent with MEN1, Mrs A had a history of primary hyperparathyroidism with persistent mild hypercalcemia despite 2 previous parathyroid explorations and a pituitary microprolactinoma, requiring cabergoline to achieve pregnancy. She had stable pancreatic lesions that were first noted on imaging in 2011 and were stable in size in 2017 and 2020, and had features consistent with benign neuroendocrine neoplasms on biopsy. She had no history of hypoglycemia, and her first pregnancy (3 years prior) was uneventful without gestational diabetes or hypoglycemia.

During this pregnancy, early screening with a 75-g oral glucose tolerance test for gestational diabetes was performed because of an elevated body mass index (BMI) and a family history of type 2 diabetes. She was diagnosed with gestational diabetes at 15 weeks’ gestation (diagnosed with an elevated fasting glucose of 97.2 mg/dL [5.4 mmol/L]). Her 1-hour glucose was 144 mg/dL (8.0 mmol/L) and 2-hour glucose 86.4 md/dL (4.8 mmol/L). She was managed with lifestyle modifications. From 18 weeks’ gestation, she reported postprandial hypoglycemia associated with diaphoresis, dizziness, and tremors, which improved with eating. Self-reported capillary blood glucose levels (BGLs) during these events ranged from 27 to 45 mg/dL (1.5-2.5 mmol/L).

## Diagnostic Assessment

Mrs A was admitted to the hospital at 18 weeks’ gestation for further evaluation. Her weight was 133 kg, height 175 cm, and BMI 43, with an unremarkable physical and neurological examination. Routine laboratory studies were normal. During her admission, she had several episodes of symptomatic hypoglycemia manifesting within 1 to 3 hours after meals. Whipple triad was confirmed with symptoms consistent with hypoglycemia (diaphoresis, dizziness, tremors), low plasma blood glucose less than 36 mg/dL (2 mmol/L), and relief of symptoms with eating. She went on to have a mixed-meal test, which confirmed hyperinsulinemic hypoglycemia. The mixed-meal test was performed as per the Endocrine Society of Australia Harmonisation of Endocrine Dynamic Testing (HEDTA) manual [3], and at 60 minutes after a meal, Mrs A became symptomatic of hypoglycemia with pathology results as follows: glucose 43 mg/dL (2.4 mmol/L), insulin 55 pmol/L (7.9 IU/L), and C-peptide 0.8 nmol/L (2.4 ng/mL). Standards for interpretation of a mixed-meal test are not well established and reference ranges are extrapolated from a 72-hour fast, with inappropriate endogenous hyperinsulinemia defined as plasma glucose less than 55 mg/dL (3 mmol/L), insulin greater than or equal to 20 pmol/L (3 IU/L), and C-peptide greater than or equal to 0.2 nmol/L (0.6 ng/mL) [3]. Mrs A's proinsulin was 53 pmol/L (7.6 IU/L) (reference range < 13.3 pmol/L/1.9 IU/L) and β-hydroxybutyrate 0.05 mmol/L (0.52 mg/dL) (reference range > 2.7 mmol/L/28 mg/dL). There was no clinical or biochemical evidence of thyroid dysfunction, growth hormone deficiency, or adrenal insufficiency, and a sulfonylurea screen was negative. Insulin antibodies were mildly elevated at 2.6 U/mL (reference range, 0-0.5 U/mL). Given her history of MEN1 and known pancreatic lesions, the primary working diagnosis was an insulinoma, and she met the diagnostic criteria for an insulinoma with a compatible clinical presentation, low BGLs, with high insulin, C-peptide, and proinsulin and absence of plasma sulfonylurea metabolites [2]. However, due to the unusual nature of her presentation during pregnancy and the absence of fasting hypoglycemia, other differentials including noninsulinoma pancreatogenous hypoglycemia syndrome and factitious hypoglycemia were considered.

## Treatment

Mrs A trialed corn starch and acarbose with improvement in symptoms; however, they were not well tolerated and ceased. She retained hypoglycemic awareness with minimal neuroglycopenic symptoms, and symptoms were managed with frequent low glycemic index foods, although the need for frequent meals resulted in Mrs A putting on 20 kg of weight through the second half of her pregnancy, greater than is recommended for her BMI. Other treatments including diazoxide, somatostatin analogues, verapamil, glucocorticoids, and workup for pancreatic surgery were considered. However, due to both the risks associated with these treatments during pregnancy and because she was able to maintain euglycemia with lifestyle changes, further treatments were not undertaken during pregnancy. Imaging was delayed until post partum.

In her third trimester, her fetus was tracking as small for gestational age (29th percentile) with decreased growth velocity. Due to fetal distress, Mrs A underwent a cesarean delivery at 37 weeks’ gestation. She had a healthy baby weighing 2.51 kg. Thirty-six hours post partum, she had one episode of asymptomatic hypoglycemia at 34 mg/dL (1.9 mmol/L) recorded on capillary BGL, with no further episodes. She was discharged home and advised to monitor her BGLs closely.

## Outcome and Follow-up

Mrs A remained well in the following months, and it was not until 8 months post partum that she reported symptomatic hypoglycemia on multiple occasions (lowest recorded capillary BGL 27 mg/dL [1.5 mmol/L]). Repeat mixed-meal test confirmed hypoglycemia (plasma glucose 46.8 mg/dL [2.6 mmol/L]) with unsuppressed insulin 83 pmol/L (12 IU/L) and C-peptide 1.3 nmol/L (3.9 ng/mL). Magnetic resonance imaging (MRI) showed stable pancreatic lesions, largest in the tail measuring 29 × 25 × 19 mm and 2 small lesions in the body measuring 9 × 8 × 7 mm and 6 × 13 × 12 mm.

**Figure 1.**
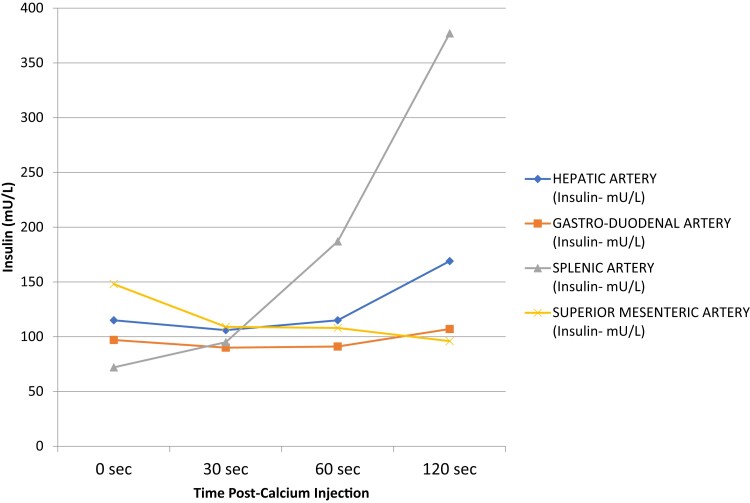
Selective arterial calcium stimulation test. The line marked with triangles demonstrates a sharp increase in insulin release from the splenic artery helping to localize the location of the insulinoma.

**Figure 2.**
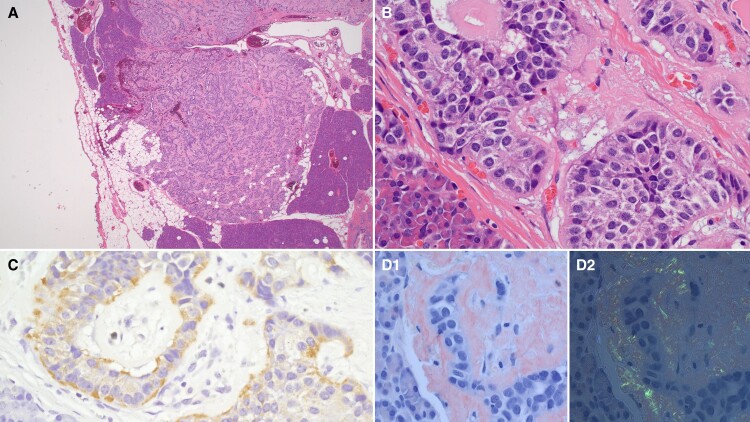
Histopathology of the largest pancreatic neuroendocrine tumor (26 mm). A, Hematoxylin-eosin (H + E) stain, low power (objective ×2); B, H + E stain, higher power (objective ×40); C, Positive immunohistochemical staining for insulin; D1, positive staining with Congo red, unpolarized; D2, Congo red, polarized with “apple green” birefringence, consistent with amyloid deposition.

A [^68^Ga] gallium-DOTA-exendin-4 positron emission tomography/computed tomography (PET/CT) did not reveal any exendin-avid pancreatic tumors. A selective arterial calcium stimulation test was performed that involved selective arteriography and infusion of calcium gluconate into the superior mesenteric, splenic, and gastroduodenal arteries with blood sampling for insulin from the right hepatic vein. The results localized the insulinoma to the body or tail of the pancreas, which are supplied by the splenic artery (Fig. 1). She underwent a laparoscopic distal pancreatectomy at 12 months post partum. Histopathology confirmed 3 benign neuroendocrine tumors measuring 26 mm, 12 mm, and 7 mm, together with 8 neuroendocrine microadenomas arising in a background of diffuse islet cell hyperplasia. The largest lesion stained for amyloid, and immunohistochemistry was positive for insulin consistent with an insulinoma (Fig. 2). After the operation, she had no further hypoglycemic episodes, and her postmeal capillary BGLs were all greater than 90 mg/dL (5 mmol/L). She lost 17 kg in weight in the first 6 months after surgery and remains well 1 year after surgery.

## Discussion

Insulinomas are rare functional neuroendocrine tumors arising from islet cells of the pancreas, and 5% to 10% are associated with MEN1 [4]. To the best of our knowledge, this is the first confirmed case of an insulinoma associated with MEN1 presenting in pregnancy.

There are 37 reported cases of insulinomas presenting in pregnancy and post partum [1, 2]. Of these, only 20% (7/36) presented in the second or third trimester. Insulinomas rarely present in pregnancy because pregnancy is an insulin-resistant state. This is secondary to placental hormones (human placental lactogen, leptin, tumor necrosis factor α) that alter maternal glucose metabolism, induce peripheral insulin resistance, and contribute to altered β-cell function [5]. In the post partum period, insulin sensitivity is quickly recovered, which, in addition to breastfeeding, can lead to hypoglycemia shortly after delivery. However, unusually, our patient presented in her second trimester, and her hypoglycemia improved initially post partum.

The diagnosis of an insulinoma is established by demonstrating inappropriate hyperinsulinemia with corresponding hypoglycemia. Although fasting hypoglycemia is more common, a subset of patients with insulinoma present with postprandial hypoglycemia [4]. In our case, Mrs A's symptoms were primarily postprandial, and hence, a mixed-meal test was performed instead of a 72-hour fast. No reference range has been established for the diagnosis of hyperinsulinemia on a mixed-meal test during pregnancy. Indeed, the consensus on reference ranges in the nonpregnant state is also not well established [3]. Although our patient had mildly elevated insulin antibodies, this was not thought to be clinically significant as people with insulin autoimmune syndrome typically have insulin antibodies 1000 times the upper limit of normal [6]. Noninvasive localization studies include CT, MRI, ^111^In-DOTA-exendin-4 single-photon emission CT (SPECT)/CT, and ^68^Ga-DOTA-exendin-4 PET/CT with increasing order of accuracy in detecting insulinomas [7]. Intravenous calcium gluconate stimulates insulin release from insulinomas, but not from normal β cells, hence a selective arterial calcium stimulation can help localize an insulinoma when imaging is negative or equivocal, or, in the setting of MEN-1, when multiple lesions are seen, it can be used to identify which of the lesions is secreting insulin. All localization studies are not without risk in pregnancy, hence, in our case, because the patient was able to manage her symptoms with dietary changes, investigations were delayed until post partum.

Insulinomas are definitively managed with surgical resection when possible. Hypoglycemia can be managed with frequent, small, low glycemic index meals as well as a number of medications such as diazoxide, somatostatin analogues, and verapamil [1]. Safety in pregnancy has not been established for these treatments. Human data on the use of diazoxide during pregnancy are limited; it has been used for the treatment of hypertensive emergencies in pregnancy during the second and third trimesters, and reportedly causes reversible alopecia or hypertrichosis in infants [8]. Somatostatin analogues such as octreotide are generally avoided in pregnancy because of risk of intrauterine fetal growth restriction [9]. Some women have been managed with continuous intravenous dextrose until delivery. Surgical removal remains the definitive treatment but is delayed until post partum if possible [1].

In the reported case studies, 34 of the 37 patients underwent surgical treatment: One-third were operated on during pregnancy and two-thirds in the postpartum period. Postoperative complications were rare. Only 2 of 34 patients showed persisting hypoglycemia requiring a second exploration. The remaining 3 patients who did not have surgery had evidence of metastatic disease and were managed with medical therapies [1]. Fetal outcomes were favorable for women diagnosed with an insulinoma during their pregnancy, with 20 of 30 neonates born at term with a mean birth weight of 3.14 kg (1.18 kg-4.15 kg) [2].

An Australian case series reported maternal MEN 1 to be associated with an increased risk of gestational diabetes, hypertensive disorders, and low neonatal birthweight, but not with an increased miscarriage rate [5]. The high rate of gestational diabetes described in this series (56%) is consistent with known high rates of type 2 diabetes among people with MEN 1 although the exact pathogenesis is unknown. Antenatal management for women with MEN1 is primarily based on data from case series with management focused on primary hyperparathyroidism and pituitary tumors in pregnancy [10]. For women with neuroendocrine tumors, like our patient, the influence of pregnancy on the evolution of neuroendocrine tumors as well as the effect of neuroendocrine tumors on pregnancy is not well described [10]. Pancreatic neuroendocrine tumors are known to express progesterone receptors and, less often, estrogen receptors, and hence neuroendocrine tumors may be sensitive to physiological changes in sex hormones during pregnancy [5]. Based on previously reported cases and our experience, we would suggest all women with MEN1 have early screening for gestational diabetes and women with known neuroendocrine tumors be monitored clinically. There is insufficient evidence to support routine pancreatic imaging preconception or during pregnancy.

We describe a patient with a history of MEN1 and known stable pancreatic neuroendocrine lesions presenting with postprandial hypoglycemia in the second trimester of pregnancy. We do not know why stable pancreatic lesions, which were quiescent for many years, became active during pregnancy, remitted for several months post partum, and later relapsed. Unusually, the patient’s frequency and severity of hypoglycemia improved post partum when insulin sensitivity is expected to recover, and surgery was delayed until 12 months post partum. Very few cases of insulinomas have presented in advanced pregnancy, and this is the only reported case of MEN1 presenting with insulinoma in pregnancy.

## Learning Points

1. Very few insulinomas present in pregnancy as the associated insulin resistance—particularly in the second and third trimester—is protective against hypoglycemia.
2. There is minimal evidence on the appropriate management of insulinomas during pregnancy. The safety of agents such as diazoxide and octreotide are not well studied in pregnancy.
3. If possible, insulinomas in pregnancy can be conservatively managed with surgical resection delayed until post partum, particularly if presenting post prandial rather than fasting hypoglycemia.

## Data Availability

Data sharing is not applicable to this article as no data sets were generated or analyzed during the present study.

## Abbreviations

BGL: blood glucose level
BMI: body mass index
CT: computed tomography
MEN1: multiple endocrine neoplasia type 1
MRI: magnetic resonance imaging
PET: positron emission tomography
